# Characterization of the most austral autochthonous dengue outbreak reported in the world (city of Bahía Blanca, Argentina, January–June 2024). A cross-sectional study

**DOI:** 10.1016/j.lana.2025.101254

**Published:** 2025-10-04

**Authors:** Guillermo Gabriel Barrenechea, Rocío Sánchez, Ornella Cynthia Calderón, Ignacio Rodrigo Buffone, Leonardo Soares Bastos

**Affiliations:** a Dirección General de Epidemiología, Ministerio de Salud, Neuquén, Argentina; b Programa de Pós-Graduação de Epidemiología em Saúde Pública - Programa Educacional VigiFronteiras, Brazil. Fundação Oswaldo Cruz, Rio de Janeiro, Brazil; c Facultad de Ciencias Naturales e Instituto Miguel Lillo, Universidad Nacional de Tucumán (UNT), Miguel Lillo 205, San Miguel de Tucumán, Tucumán CP 4000, Argentina; d Departamento de Epidemiología y Calidad, Secretaría de Salud de Bahía Blanca, Buenos Aires, Argentina; e Fundação Oswaldo Cruz, Presidência, Programa de Computação Científica, Rio de Janeiro, Brazil; f Instituto de Matemática Pura e Aplicada e Tecnológica (IMPA Tech), Rio de Janeiro, Brazil

**Keywords:** Bahía Blanca, Dengue, Outbreak

## Abstract

**Background:**

Dengue is a vector-borne viral disease that is expanding its boundaries, causing outbreaks and autochthonous viral circulation in places where it had not previously been recorded. The aim is to describe epidemiologically the first outbreak of dengue in the southernmost latitude of the planet ever recorded.

**Methods:**

In Bahía Blanca (Buenos Aires, Argentina), dengue virus circulation was reported between January 1, 2024, and June 10, 2024. Cases were detected and reported to the Health Secretariat of the Municipality of Bahía Blanca, Province of Buenos Aires, Argentina. The cases were clinically diagnosed and tested positive for dengue virus (DENV) nonstructural protein 1 (NS1), RT-PCR, and/or IgM. Cases were classified as autochthonous when patients did not report traveling to areas with dengue circulation during the 15 days prior to the onset of symptoms. All serological and molecular analyses were performed at the Municipal Hospital. This study was conducted using clinical samples and data obtained during the outbreak, and all personal identifiers were excluded.

**Findings:**

A total of 94 positive cases were reported out of 470 suspected cases. Of the total confirmed cases, 63 were classified as autochthonous and 28 as imported. Serotypes DENV1 and DENV2 were detected in both cases. The first autochthonous case was identified in the second epidemiological week, and the peak of the epidemic curve occurred in the thirteenth epidemiological week. Twenty-seven imported cases came from Argentine locations with autochthonous viral circulation, and one came from Paraguay.

**Interpretation:**

This study provides clear evidence of the expansion of dengue fever to latitudes that were not included in previously published risk maps for Argentina. Reporting on the expansion of dengue fever to new areas should alert decision-makers to adopt public health policies aimed at reducing the burden of the disease.

**Funding:**

LSB, GGB, RS were partially funded by the call 18/2023 by the 10.13039/501100003593National Council for Scientific and Technological Development (CNPq) and the Department of Science and Technology of Secretariat of Science, Technology, Innovation and Health Complex of 10.13039/501100006506Ministry of Health of Brazil (DECIT/SECTICS/MS). LSB also acknowledges research grants from 10.13039/501100004586FAPERJ (E-26/201.277/2021) and 10.13039/501100003593CNPq (310530/2021-0).

**Research in context**

**Evidence before this study**

Dengue is a vector-borne viral disease that is expanding its frontiers, causing outbreaks and autochthonous viral circulation in places never reported before. Dengue disease is endemic in more than 100 countries and is spreading to new areas in Europe, the eastern Mediterranean and South America. The dengue distribution boundary can be approached according to political divisions, for example, within the same country, between countries or on a more regional scale. However, the expansion of dengue refers to its distribution in latitudinal terms and on a global scale. In Argentina, in the last decade, the distribution of dengue has expanded westward and southward following a pattern of new isolated records rarely exceeding 37°S. In the same direction, from north to south and from east to west, reports of new areas of autochthonous circulation also began. The first outbreaks of considerable scale, after the reintroduction of dengue in Argentina, were reported mainly in the northern part of Argentina, during 2009, 2016 and 2020. In Argentina, DENV is transmitted mainly by *Aedes aegypti* whose distribution reaches tropical and subtropical regions of the Americas. There are occasional reports of vector presence in the northern Patagonian region of Argentina but previous publications of models of future dengue distributions do not include this region. We describe the first dengue outbreak reported in a temperate city in Argentina that shows the most extreme virus distribution reported so far.

**Added value of this study**

This first record of dengue disease in a temperate city of the southern cone provides evidence of an expansion of the virus towards latitudes that escaped previously published risk maps for Argentina. In this study, between January 1 and June 10 of 2024, we reported a total of 94 positive cases out of 470 suspected cases in Bahía Blanca, a temperate city in the southern region of Argentina. Of the total number of confirmed cases, 63 were classified as autochthonous and 28 as imported. Serotypes DENV1 and DENV2 were found in both cases. At the temporal level, the distribution of autochthonous cases began in the 6th epidemiological week of 2024, with the peak of the epidemic curve in the 13th epidemiological week with a total incidence was 27.9 cases/100,000 population. Most of the autochthonous cases were in the urban area, predominantly in the northwestern sector of the city. We described a very detailed outbreak in terms of magnitude, variety of circulating serotypes and extreme geographical situation. This study is clear evidence of the expansion of dengue and a starting point to test different hypotheses that may be influencing the spread of the disease to extreme latitudes.

**Implications of all the available evidence**

Our findings confirm the value and importance of epidemiological and entomological surveillance. Reporting the expansion of dengue to new areas, must alert decision makers to take public health policies to reduce the burden of the disease. Surveillance of imported cases is crucial to identify and size the influx of people with dengue disease who may be acting as potential triggers of an outbreak. The presence of the vector in the area should warn the region of the possibility of cryptic circulation of DENV, which may have caused asymptomatic infections. In fact, secondary DENV infections have been reported in the city of Bahía Blanca in areas considered as non-endemic. Since the reintroduction of dengue in Argentina, it has not been enough time to state that dengue reached these latitudes only because of climate change, but we must broader the spectrum and include other fields in the model development to explain the rapid spread. It is essential to use and integrate different quantitative approaches incorporating data not only from climate but also from other fields related to the effect of the Anthropocene and check if their incorporation improves the predictions of the spread of dengue at a global level.

## Introduction

Dengue is a vector-borne viral disease that is expanding its boundaries, causing outbreaks and autochthonous viral circulation in places where it had never been recorded before. It is the most important cause of arbovirus disease worldwide, with a dramatic increase in incidence in recent decades.^1^^,^^2^ In 2023, it reached a historic peak with more than 6.5 million cases and more than 7300 deaths, affecting more than 80 countries worldwide. From January to September 2024, these figures have already been exceeded, with more than 12 million cases and 8700 deaths.^3^ The disease is endemic in more than 100 countries and is spreading to new areas of Europe, the eastern Mediterranean, and South America.^4^^,^^5^ The distribution boundary of dengue can be addressed according to political divisions, for example, within a single country, between countries, or on a more regional scale. However, the expansion of dengue refers to its distribution in latitudinal terms and on a global scale. In this context, previous publications of future dengue distribution models do not include the southern boundary of the American continent.^6^, ^7^, ^8^

The spread of the dengue virus requires primary and secondary hosts. In the case of the mosquito vector, its presence or distribution is determined by its ability to reach a given location, establish itself, and perpetuate itself through reproduction, like any living being.^9^^,^^10^ In the case of the vertebrate host, humans, its presence or distribution in a given location is determined by a combination of ecological, social, and economic factors, depending on social policies and regulations. In this sense, the new Anthropocene era is characterized by three fundamental issues that are directly related to dengue: climate change, human mobility, and changes in landscape epidemiology.^11^^,^^12^ Climate change can provide new niches for the establishment of the vector, thus generating its expansion into new areas. Human mobility can act as an agent for spreading the disease to new locations, minimizing distances and accelerating its arrival to potentially susceptible populations. Finally, landscape modification, changes in land use, and growth and development offer new niches for dengue vector mosquitoes. The interaction and synergy between these three factors could accelerate the spread of the disease predicted by current predictive models.^13^

In Argentina, the first autochthonous cases of dengue were reported in 1905.^14^^,^^15^ Although it had been eradicated 1963, in 1986 a reinfestation was reported in the northern region.^16^ In less than 10 years (1991), the northern part of the province of Buenos Aires (32° south latitude) reported the presence of dengue and in 1995, it established itself in the Federal Capital. In 2000, the southern distribution range extended across Argentina from the northwest to the southeast, with positive locations at latitudes up to 35°S. In the last decade, the distribution has expanded westward and southward, following a pattern of new isolated records that rarely exceed 37°S.^17^^,^^18^ In the same direction, from north to south and east to west, reports of new areas of autochthonous circulation also began to appear.^19^^,^^20^ The first outbreaks of considerable magnitude, following the reintroduction of dengue in Argentina, were reported mainly in the northern part of the country, during the years 2009, 2016 and 2020.^21^^,^^22^

In order to increase evidence of this global expansion of dengue, our aim is to describe the epidemiology of the first outbreak recorded to date in the southernmost latitude of the planet. This work will be the starting point for testing different hypotheses that may be influencing the spread of the disease to extreme latitudes.

## Methods

### Scope of study

**Fig. 1:**
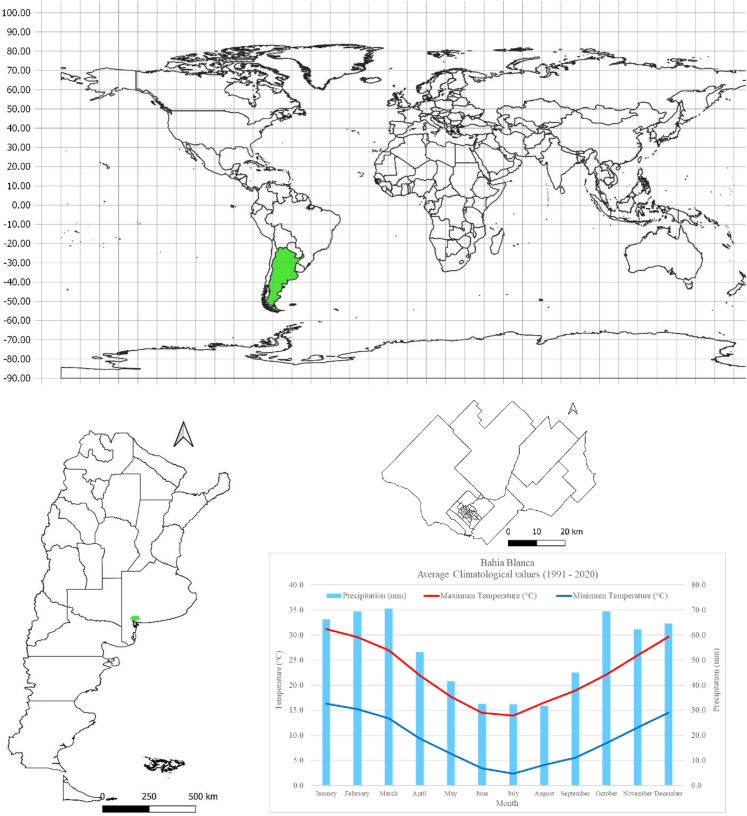
Above. Detail of Argentina’s geographical location. Below: Location of the city of Bahía Blanca within the province of Buenos Aires (green point) with details of Bahía Blanca and its climogram.

Bahía Blanca, the main city in the district of the same name, is located at 38° 44’ south latitude and 62° 16' west longitude, about 630 km from the country’s capital. Geographically, it is located in the southwest of the province of Buenos Aires, Argentina, one of the southernmost countries in the world. The city occupies a strategic position within the province of Buenos Aires. It has the deepest port in South America, is home to a large petrochemical center, and is surrounded by agricultural and livestock production areas. This makes it a city with a large movement of people, as it is connected by land to the provincial capital (the city of La Plata) and by land and air to the country’s capital. Bahía Blanca has a population of 336,571 inhabitants.^23^ The climate is semi-arid, with a transition between the hot and humid climate of eastern Buenos Aires province and the cold and dry climate of Patagonia. It is characterized by marked seasonal temperature variations that distinguish between summer and winter.^24^ According to data provided by the National Meteorological Service for the period 1991–2020, the city of Bahía Blanca has an average annual temperature of 15.41 °C; the average annual precipitation is 639.10 mm, with a high annual variation, with more precipitation in late spring and early summer (Fig. 1). Winds are moderate and predominantly from the north-northwest, with average maximum speeds of 70 km/h (Fig. 1).

### Study design

This is an outbreak study that analyzes dengue cases that occurred in the city of Bahía Blanca, Buenos Aires province, Argentina, between January and June 2024. We followed the Strengthening the Reporting of Observational Studies in Epidemiology (STROBE) checklist for cross-sectional studies. Descriptive statistics were used to create tables, graphs, and maps. Summary measures were calculated, and proportions were compared as part of the analysis. A Pearson chi-square test was performed to assess whether the proportion of patients with and without symptoms varied depending on whether they were autochthonous or imported. To this end, autochthonous cases were separated from imported cases and compared with each other according to the proportions with and without symptoms (the most frequent were considered), respectively. The software used was R and Qgis.^25^^,^^26^

### Case definition

Cases were considered positive when clinically diagnosed and tested positive for DENV nonstructural protein 1 (NS1) and/or IgM, and some of them underwent real-time reverse transcription polymerase chain reaction (RT-PCR), which allowed the DENV type to be identified. Cases were classified as autochthonous when patients reported no travel to areas of circulation during the 15 days prior to the date of symptom onset (date of onset).

At the peak of the outbreak, cases were confirmed using clinical epidemiological criteria, considering a positive laboratory case in the same family group, cohabitant, or if another case was verified within the household. The date of symptom onset was always considered. Cases were considered suspected or probable when they involved a person who lived in or had traveled in the last 14 days before the onset of symptoms to areas with dengue transmission or vector presence, and had acute fever, usually lasting 2–7 days, and two or more of the following manifestations: nausea or vomiting, rash, headache or retro-orbital pain, myalgia or arthralgia, petechiae, or positive tourniquet test (+), leukopenia with or without warning signs or manifestations of severity.

### Ethical approval

This study has used anonymized data that does not process any sensitive individual or personal data. Therefore, this study does not require any ethical approval.

### Role of the funding source

LSB, GGB, RS were partially funded by the call 18/2023 by the National Council for Scientific and Technological Development (CNPq) and the Department of Science and Technology of Secretariat of Science, Technology, Innovation and Health Complex of Ministry of Health of Brazil (DECIT/SECTICS/MS). LSB also acknowledges research grants from FAPERJ (E-26/201.277/2021) and CNPq (310530/2021-0). The funders had no role in the study design, data collection and analysis, preparation of this manuscript, interpretation of results, or the decision to submit the manuscript for publication.

## Results

**Fig. 2:**
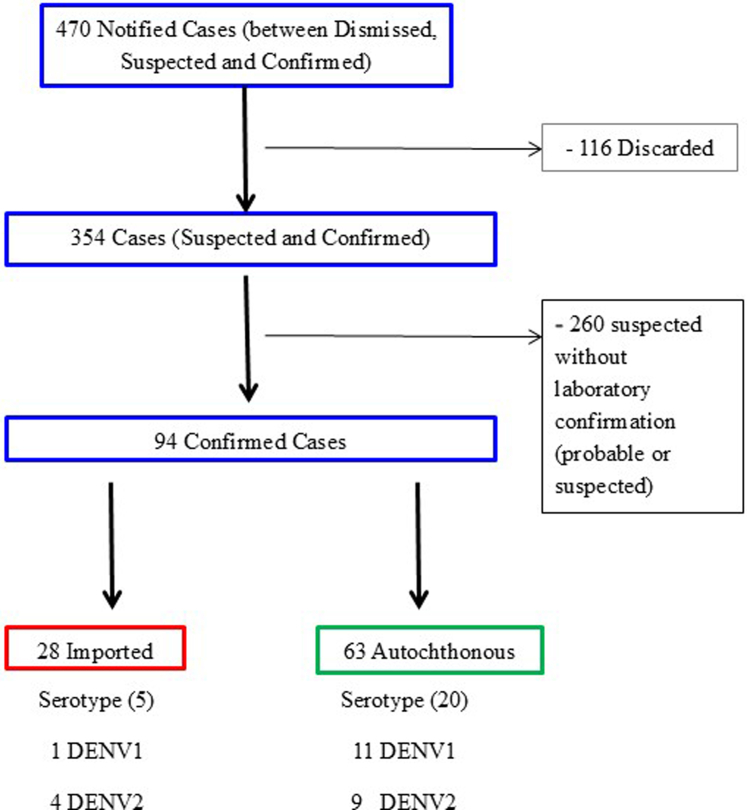
Detail of the epidemiological course of dengue fever from the appearance of the first suspected case to the identification of the serotypes of confirmed cases. City of Bahia Blanca. Period 2024.

Between January 1 and June 10, 2024, a total of 470 suspected cases of dengue were reported to the Health Secretariat of the municipality of Bahía Blanca (Buenos Aires province, Argentina). Of these, 94 were confirmed by laboratory tests, of which 28 were classified as imported dengue cases and 63 as autochthonous dengue cases (Fig. 2). The remaining three cases could not be classified correctly due to a lack of accurate information. No deaths were reported.

The average age of patients confirmed with dengue classified as imported and autochthonous was 35.9 years (standard deviation of 15.3 years). In terms of sex, no obvious differences were observed in the proportion of men and women in imported cases (13 female cases/14 male cases) or in autochthonous cases (33 female cases/28 male cases). Information on the sex of some individuals was not available. Signs and symptoms did not show significant differences (Table 1) between autochthonous and imported cases.

**Table 1:** Description of frequency of most frequent symptoms and their comparison between autochthonous and imported cases of dengue fever.

Symptoms	Imported cases (N = 28)		Autochthonous cases (N = 63)		Pearson’s Chi-squared test
	Presence	Absence	Presence	Absence	*p*-value
Fever higher than 38 °C	24	4	52	11	0.53
Headaches	22	6	49	14	1.00
Myalgias	19	9	44	19	1.00
Arthralgias	11	17	37	26	0.14
Retroocular pain	12	16	24	39	0.84
Abdominal pain	6	22	18	45	0.65
Nauseas/vomiting	8	20	23	40	0.62
Diarrhea	9	19	18	45	0.92
Rash	2	26	16	47	0.08
Bleeding	1	27	1	62	1.00

Of the imported cases, 23 out of 28 originated in different locations in Argentina where autochthonous circulation of the virus had already been declared. Only one case was confirmed as originating in Paraguay, a neighboring country of Argentina (Table 2).

**Table 2:** Details of the sources of the imported cases of dengue fever in Bahía Blanca, 2024.

Id	Origin Location	Frequency^a^ (*n* = 24)	Distance from Bahía Blanca (Km)	Access way	
1	CABA	5	636.6	Land/Air	
2	Misiones	4	1743.2	Land	
3	Salta	4	1817.1	Land	
4	Córdoba	3	938.2	Land	
5	Corrientes	2	151.9	Land	
6	Santa Fé	2	950.2	Land	
7	Chaco	1	1598.2	Land	
8	Tucumán	1	1504.5	Land	
9	La Pampa	1	434.6	Land	
10	Paraguay	1	1868.6	Land	

Id is the code used to identify each jurisdiction on the map and Frequency is the absolute frequency of confirmed cases that came from those jurisdictions.

CABA: Ciudad Autónoma de Buenos Aires.

a Data were only available for 24 of the 28 imported cases.

The total number of cases corresponded to people who apparently had the disease for the first time. The serotype could be verified in 18% (5/28) of imported cases, with 80% DENV2 (4/5). Among autochthonous cases, the serotype was identified in 32% (20/63), with 55% DENV1 (11/20) (Fig. 2).

**Fig. 3:**
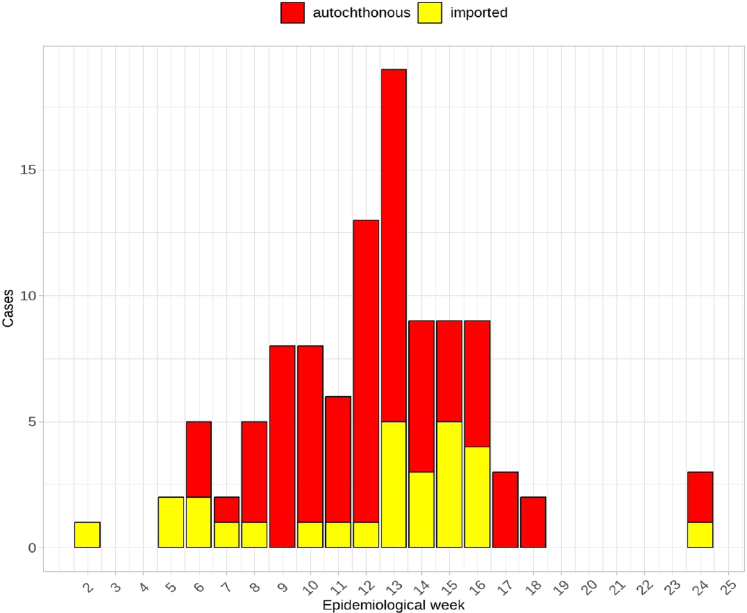
Epidemic curve of the first dengue outbreak in Bahía Blanca, 2024.

At the temporal level, the distribution of autochthonous cases began in the 6th epidemiological week of 2024 (February 8), with the peak of the epidemic curve occurring in the 13th epidemiological week (March 26) (Fig. 3). The total positivity rate recorded in the outbreak was 94 out of 470 (20%), and the total incidence was 27.9 cases/100,000 inhabitants.

**Fig. 4:**
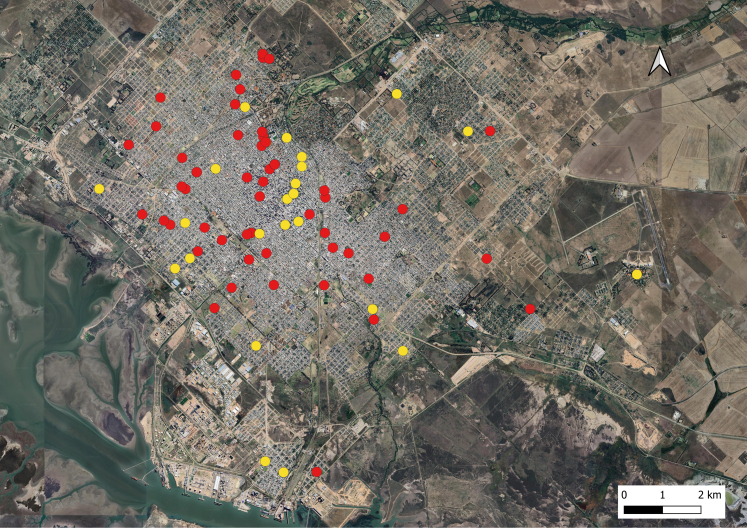
Distribution of reported cases reported in the dengue outbreak in Bahía Blanca, according to autochthonous (red) and imported (yellow) cases (period: from January 1 to June 10, 2024).

Spatially (Fig. 4), most of the autochthonous cases were recorded in the urban area, predominantly in the northwestern sector. Approximately 98% (62/63) of them occurred in the urban area of the main city of Bahía Blanca, although one of them occurred in the town of “Gral. Daniel Cerri”, which belongs to the district of Bahía Blanca. Cases occur in all areas of the urban area of the city.

## Discussion

This article describes the first dengue outbreak in the city of Bahía Blanca, Buenos Aires province, Argentina, which was the southernmost case reported to date. The description of the outbreak shows the co-circulation of two serotypes (DENV1 and DENV2) with a discrepancy in the prevalence of serotypes between imported and autochthonous cases.

Reporting on the spread of dengue to new areas, such as Bahía Blanca, should alert decision makers to act quickly to reduce the burden of the disease. Although dengue has no specific treatment, it is important that healthcare personnel are well trained to diagnose and treat a case of dengue, as it could progress to a severe case.^27^^,^^28^ Another important public health measure in an emerging area is vector control, which is challenging, but if the mosquito population is not large, traditional methods of larval and adult control can be effective.^29^

There are several reports of dengue outbreaks in high latitudes in the north hemisphere.^30^, ^31^, ^32^ However, very few studies describe the outbreak in detail in terms of magnitude, variety of circulating serotypes and extreme geographical location.

In Argentina, DENV is mainly transmitted by *A. aegypti*, which is distributed throughout the tropical and subtropical regions of the Americas. In Argentina, there are occasional reports of the vector’s presence in the northern region of Patagonia, and since 2009, its presence has been recorded in the city of Bahía Blanca.^33^^,^^34^ The presence of the vector in the area should alert the region of the possibility of cryptic circulation of DENV, which could have caused asymptomatic infections. In fact, secondary DENV infections have been reported in the city of Bahía Blanca in areas considered non-endemic.^20^

Recent literature supports the idea that the spread of dengue does not depend exclusively on intrinsic factors of the vector and the host, but also on other factors associated with anthropogenic activity. In this context, climate, human mobility, and landscape epidemiology can determine the current and future epidemiological scenarios of dengue. Temperature influences the colonization and annual persistence of *A. aegypti* populations.

The geographical distribution of the vector would require a winter isotherm of 10 °C; however, it has been suggested that an average annual temperature of 15 °C would be a better predictor for its distribution.^35^ The city of Bahía Blanca has an average annual temperature of 15.41 °C, according to SMN (1991–2020 period), which would be within the appropriate areas the establishment and persistence of the vector. Colder areas may offer places where eggs could hatch but not produce viable larvae (places unsuitable for colonization), while other places may provide suitable conditions for the development of viable larvae during the spring.^36^ Climate variability could increase average annual temperature values, shifting the vector’s distribution limits toward higher latitudes.^37^

Although climate plays an important role, why is there still no autochthonous viral circulation in cities where the vector is present and climatic conditions are similar? Increased human mobility between cities may be an important factor to consider. In the case of Bahía Blanca, most of the imported cases during the outbreak came from Autonomous City of Buenos Aires (CABA), a city located in the same province, Buenos Aires. In this context, it would be interesting to study the connection routes between the population of Bahía Blanca and its region, which could contribute to the understanding the spread of the virus.^38^

### Limitations of the study

The limitations of an observational study include an underestimation of the outbreak due to the number of cases reported. One possibility is that, as this was the first outbreak, the health system’s alert level did not allow for more active surveillance to detect a greater number of cases. Furthermore, this study does not present information on cases that may have been reported in the private health sector. These could be the reasons for the underestimation of the outbreak. However, the health system was sufficiently alert and responsive to resolve the cases that were reported. This helped to establish the first outbreak in the region with accurate information. This is useful not only locally, but also globally, as it allows us to understand a little more about the dynamics of the disease and what we can expect in the coming years.

Another limitation of the study is that it did not evaluate the detection of the dengue virus in mosquitoes.

### Conclusion

This study describes the first recorded outbreak of dengue fever in Bahia Blanca, a temperate city of the southern cone. Reports of dengue spreading to new areas, such as Bahía Blanca, should alert policymakers to quickly adopt public health measures aimed at reducing the burden of the disease.

Surveillance of imported cases is crucial to identify and quantify the influx of people with dengue who may act as potential triggers for an outbreak.^39^ This first record and its epidemiological description are very important because they provide evidence of the expansion of the virus to higher latitudes. In fact, in Argentina, published risk maps showed no evidence of expansion to the latitudes found in this study. In this context, the findings presented here may serve as an impetus for further progress, in the sense that the behavior of an event cannot be modeled from a single perspective or by focusing on a single source. Since the reintroduction of dengue in Argentina, not enough time has passed to say that dengue reached these latitudes solely due to climate change, but we must broaden the spectrum and include other fields in the development of the model to explain the rapid spread. It is essential to use and integrate different quantitative approaches that incorporate data not only from climate, but also from other fields related to the Anthropogenic Global Warming Effect, and to verify whether their incorporation improves predictions of the spread of dengue worldwide.

## Contributors

- GGB. Conceptualization, data curation, formal analysis, methodology, supervision, validation, visualization, writing—original draft, and writing—review & editing. Directly accessed and verified the underlying data reported in the manuscript and are responsible for the decision to submit the manuscript.
- RS. Access to all the data reported in the study. Accessed and verified the data.
- OCC. Access to all the data reported in the study. Accessed and verified the data.
- IRB. Access to all the data reported in the study. Accessed and verified the data.
- LSB. conceptualization, funding acquisition, methodology, resources, validation, writing—original draft, and writing—review & editing.

## Data sharing statement

The datasets generated and analyzed during the current study are not publicly available. They are available from the corresponding author on reasonable request and following approval from the Municipal Secretariat of Health of Bahía Blanca.

## Editorial disclaimer

The Lancet Group takes a neutral position with respect to territorial claims in published maps.

## Declaration of interests

We declare no competing interests.

## Appendix A Supplementary data

Translated Abstract
