# Supplementary material for: Characterization of the most austral autochthonous dengue outbreak reported in the world (city of Bahía Blanca, Argentina, January–June 2024). A cross-sectional study
Source: Lancet Reg Health Am. 2025 Oct 4;51:101254. doi: 10.1016/j.lana.2025.101254 (PMC12528942; doi:10.1016/j.lana.2025.101254)
Supplement: Translated Abstract [file mmc1.docx]

**Editorial disclaimer**

The translation of the Summary was submitted by the authors, and we reproduce it as supplied. It has not been peer reviewed. Our editorial processes have only been applied to the original version in English, which should serve as a reference for this manuscript.

**Resumen (250 palabras)**

***Introducción***

El dengue es una enfermedad viral transmitida por vectores que está traspasando sus límites, provocando brotes y circulación viral autóctona en lugares no reportados anteriormente. El objetivo es describir epidemiológicamente el primer brote de dengue jamás reportado en la latitud más austral del planeta.

***Métodos***

En Bahía Blanca (Buenos Aires, Argentina) se reportó circulación viral de dengue entre el 1 de enero de 2024 y el 10 de junio de 2024. Los casos fueron detectados y notificados a la Secretaría de Salud del Municipio de Bahía Blanca, Provincia de Buenos Aires, Argentina. Los casos fueron diagnosticados clínicamente y fueron positivos para la proteína no estructural 1 (NS1) del virus del dengue (DENV), RT-PCR y/o IgM. Los casos se clasificaron como autóctonos cuando los pacientes informaron que no habían viajado a áreas de circulación de dengue durante los 15 días anteriores a la fecha de aparición de los síntomas. Todos los ensayos serológicos y moleculares se realizaron en el Hospital Municipal de la ciudad de Bahía Blanca. Este estudio se realizó utilizando muestras clínicas y datos obtenidos durante el brote y se excluyeron todos los identificadores personales.

***Resultados***

Se reportaron un total de 94 casos positivos de 470 casos sospechosos. Del total de casos confirmados, 63 fueron clasificados como autóctonos y 28 como importados. En ambos casos se encontraron serotipos DENV1 y DENV2. El primer caso autóctono se identificó en la 2ª semana epidemiológica y el pico de la curva epidémica se produjo en la 13ª semana epidemiológica (. Del total de casos importados, 27 provenían de localidades argentinas con circulación viral autóctona y uno provino de Paraguay.

***Interpretación***

Este trabajo es una clara evidencia de la expansión del dengue hacia latitudes que escaparon a los mapas de riesgo publicados previamente para Argentina. Informar sobre la expansión del dengue a nuevas áreas debe alertar a los tomadores de decisiones para que adopten políticas de salud pública que reduzcan la carga de la enfermedad.

***Fondos***

LSB, GGB, RS fueron parcialmente financiados por la convocatoria 18/2023 del Consejo Nacional de Desarrollo Científico y Tecnológico (CNPq) y el Departamento de Ciencia y Tecnología de la Secretaría de Ciencia, Tecnología, Innovación y Complejo de Salud del Ministerio de Salud de Brasil (DECIT/SECTICS/MS). La LSB también reconoce becas de investigación de la FAPERJ (E-26/201.277/2021) y del CNPq (310530/2021-0).
